# tRNA-derived small RNAs (tsRNAs) in cardiovascular diseases: biogenesis, functions, and therapeutic targets

**DOI:** 10.3389/fcvm.2025.1622248

**Published:** 2025-08-21

**Authors:** Chunfeng Xie, Sihong Chen, Chengyuan Wang, Wei Si, Zidan Wang, Qi Luo, Chan Qi

**Affiliations:** ^1^ Queen Mary School, Jiangxi Medical College, Nanchang University, Nanchang, Jiangxi, China; ^2^ School of Pharmacy, Jiangxi Medical College, Nanchang University, Nanchang, Jiangxi, China; ^3^ The Second Affiliated Hospital, Jiangxi Medical College, Nanchang University, Jiangxi, China; ^4^ School of Basic Medical Sciences, Jiangxi Medical College, Nanchang University, Nanchang, Jiangxi, China; ^5^ The First Hospital of Nanchang, The Third Affiliated Hospital of Nanchang University, Nanchang University, Nanchang, Jiangxi, China

**Keywords:** tRNA-derived small RNAs, cardiovascular diseases (CVDs), biogenesis, classification, therapeutic targets

## Abstract

tRNA-derived small RNAs (tsRNAs) are a class of non-coding RNAs that are generated by cleavage of precursors or mature tRNAs under stress conditions such as hypoxia, oxidative stress and nutrient deficiency. Recent breakthroughs in RNA sequencing technology have revealed their association with cardiovascular diseases (CVDs), including myocardial infarction (MI), atherosclerosis, cardiac hypertrophy, aortic coarctation, and pulmonary arterial hypertension. tsRNAs play important biological functions in these diseases, including the inhibition of apoptosis, epigenetic modification, intercellular signaling mediation, translation, and regulation of gene expression. In addition, tsRNAs show promise as both detectable indicators and intervention targets for CVD. This review examines the biogenesis, classification, and multifaceted functions of tsRNAs in CVD, emphasizing their dual roles as diagnostic tools and therapeutic targets. future research should focus on elucidating tsrna-mediated regulatory networks and developing RNA-based interventions to address unmet needs in cardiovascular medicine.

## 1 Introduction

Transfer RNAs (tRNAs) represent a class of compact non-protein-coding RNA (70–90 nt), that predominantly adopted into L-shaped spatial configuration. Their canonical function involves facilitating the precise incorporation of amino acid residues into growing polypeptide chains according to messenger RNA templates (1). Beyond this classical role, tRNAs can be cleaved into tRNA-derived small RNAs (tsRNAs), initially misclassified as random degradation byproducts (2). Unlike other small non-coding RNAs, tsRNAs are generated through precise cleavage of mature or precursor tRNAs by endonucleases such as angiogenin (ANG,exclusively referring to angiogenin), exhibiting exceptional stability due to their heavily modified tRNA origins (3–5).

Initial identification of tsRNA occurred during analysis of malignant disease patients' urinary specimens in the 1970s (6), yet their biological significance remained overlooked until 2009, when Fu et al. identified stress-induced tsRNAs (tiRNAs) produced via ANG-mediated cleavage (7). Advances in sequencing technologies, including PANDORA-seq and CPA-seq, enabled comprehensive profiling of tsRNA expression across diverse tissues and cell types (8, 9), revealing their evolutionary conservation and tissue-specific expression patterns (5).

Functionally, tsRNAs regulate genetic expression through transcriptional, post-transcriptional, and epigenetic levels (10, 11), modulating stress responses, gene silencing, translation control, cell cycle progression, and apoptosis (12, 13). Notably, their involvement in hypoxia and other stress responses (14) highlights their role in CVDs. Emerging evidence links dysregulated tsRNA profiles to pathological processes such as cardiac hypertrophy and ischemia-reperfusion injury. tsRNAs serve as both molecular indicators and therapeutic candidates in CVD management, offering novel strategies for disease intervention (15). Their remarkable stability in plasma and exosomes positions them as promising tools for early disease detection and prognosis, while their participation in intercellular signaling underscores their regulatory significance (16, 17). Furthermore, their ability to modulate critical cellular processes suggests therapeutic potential in mitigating myocardial injury, preventing plaque rupture, and reducing fibrosis (18).

Thus, this review summarizes the biogenesis and fundamental functions of tsRNAs, particularly emphasizing their biological significance and mechanistic implications in CVDs. A deeper understanding of tsRNA-mediated regulatory networks could improve pathophysiology and provide a theoretical foundation for innovative therapeutic approaches aimed at improving clinical outcomes.

## 2 Article types

This manuscript is a Review article prepared in accordance with the requirements outlined for Review articles on the Frontiers in Cardiovascular Medicine journal website.

## 3 Classification and discovery of tsRNAs

tsRNAs are a class of small non-coding RNAs (sncRNAs) with 18–40 nucleotides lengths that result from precise cleavage of precursor or mature tRNAs (4). According to variations in their biogenesis patterns and structural characteristics, tsRNAs are classified into two groups: tRNA-derived fragments (tRFs) and tRNA-derived stress-induced RNA (tiRNA) (19). tRFs originate from specific cleavage sites in mature or precursor tRNAs, while tiRNAs form when stress cleaves the mature tRNA anticodon loop (19). This cleavage process is highly environmentally dependent. Ribonucleases like ANG, Dicer, and ribonuclease Z/P recognize and cleave tRNA molecules at different sites under various physiological or pathological conditions (20–22).

**Figure 1:**
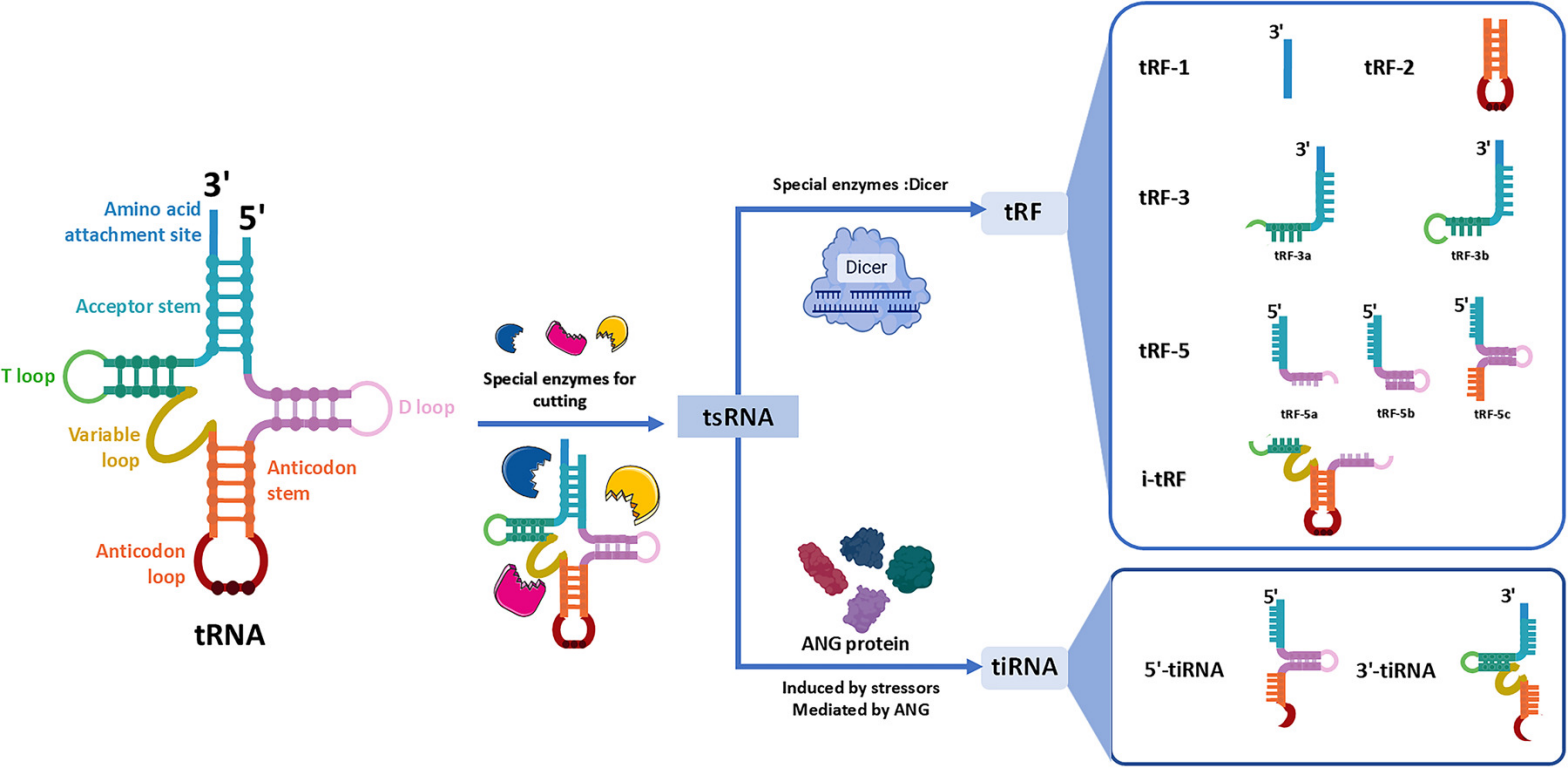
Classification of tsRNAs based on cleavage sites and enzymatic sources. tsRNAs are generated from precursors or mature tRNAs via cleavage by specific enzymes. On the left, a canonical tRNA structure is shown, including key regions such as the acceptor stem, D loop, anticodon loop, and T loop. In the middle, cleavage by enzymes such as Dicer and angiogenin (ANG) yields distinct tsRNA subclasses. On the right, tRNA-derived fragments (tRFs) are divided into five main types: tRF-1, tRF-3, tRF-5, tRF-2, and i-tRF (internal fragments covering the anticodon region). Stress-induced tiRNAs are generated through enzymatic processing by ANG at the anticodon region, yielding both 5’- and 3’-derived tiRNA species. Each subclass varies in length and biogenesis pathway, reflecting its unique functional potential in cellular processes.

Initially viewed as tRNA degradation byproducts (23, 24). However, recent studies have overturned this notion, revealing that tsRNAs can regulate gene expression through interaction with target genes. They are involved in diverse physiological processes, including cellular multiplication, motility, apoptosis, and phenotypic transformation (25). Substantial evidence also links their dysregulation with the progression of CVDs and other conditions (26). As research progresses, tsRNAs are gradually emerging as potential new targets for disease diagnosis and therapy, offering promising prospects for their clinical application (20) (Figure 1).

### 3.1 tRFs: tRNA-derived fragment

tRFs are 14–30 (nt) fragments and most tRFs are produced by precise cleavages at various locations within mature tRNA molecules. In some cases, tRFs are generated by the Dicer-mediated degradation of misfolded pre-tRNAs, a mechanism that ensures the systematic removal of aberrant tRNA species (27). Based on cleavage sites, tRFs can be systematically categorized into five main subclasses: (1) tRF-1: ribonuclease Z-cleaved 3' trailer sequences from pre-tRNAs, containing the poly-U tract. (2) tRF-3: 3’-derived fragments, including the CCA sequence generated by ANG, Dicer, or other ribonucleases. Based on their length, tRF-3 fragments are further subdivided into tRF-3a (18 nt) and tRF-3b (22 nt) (28, 29). (3) tRF-5: Produced from the 5' end of mature tRNAs, generated through enzymatic cleavage occurring either within the D-loop or at the junction between the D-loop and the anticodon loop. These fragments vary in length and are categorized into three subtypes: tRF-5a (14–16 nt), tRF-5b (22–24 nt), and tRF-5c (28–30 nt) (4, 30). (4) tRF-2: Generated from the anticodon loop of specific tRNAs, such as tRNATyr, tRNAGly, tRNAAsp, or tRNAGlu. These fragments retain portions of the double-stranded region and the anticodon loop but lack both the 5' and 3' termini of the original tRNA molecule (4, 30). (5) i-tRFs: Internal fragments that do not correspond to the traditional ends of tRNAs but are derived from internal sequences, often spanning the anticodon loop or other internal regions of tRNA (31). i-tRF is divided into 3 types: A-tRF, V-tRF, and D-tRF, which contain structures of anticodon rings, variable rings, and D-rings, respectively (32, 33).

### 3.2 tiRNAs: stress-induced tRNA halves

tiRNAs (31–40 nt) as longer fragments, generated through the cleavage of mature tRNAs at the anticodon loop. This cleavage is induced by various stressors, such as oxidative damage, oxygen deprivation, viral invasion, or thermal stress. In mammalian cells, the enzyme ANG serves as the primary mediator of this process, also along with RNase T2 and RNase L (3, 7, 34–36). Based on their biogenesis and structural characteristics, tiRNAs can be categorized into two distinct subtypes: (1) 5'-tiRNAs: These fragments originate from the 5' terminus of mature tRNAs and terminate at cleavage sites within the anticodon stem-loop (37).The formation of 5'-tiRNAs often occurs in regions of tRNA with abnormal methylation (38). It has been confirmed to be unrelated to most extracellular vesicles and is transported in the blood in the form of a complex (39). (2) 3'-tiRNAs: These fragments span from the cleavage site of the anticodon loop to the 3' terminus of the tRNA, encompassing the CCA trinucleotide sequence (37).

Comparisons between the two types of tiRNAs reveal a distinct role for 5'- and 3'-tiRNAs. Only 5'-tiRNAs can inhibit protein translation, while 3'-tiRNAs lack this function. This may be due to the additional terminal hydroxyl group and the V region present at the extra end of 3'-tiRNAs after cleavage (40). It has been pointed out that 5'-tiRNAs participate in angiogenesis and the regulation of inflammatory genes by inducing histone modifications (41). Based on the classification and functional characteristics of tiRNAs described above, our findings provide preliminary insights for further exploration of the expression patterns, molecular mechanisms, and clinical translational potential of specific tsRNAs in cardiovascular diseases (CVD). These discoveries also offer novel molecular targets for the diagnosis and treatment of cardiovascular disorders.

Exposure to stress results in a upregulation of tiRNAs fragment. In experimental models of renal ischemia/reperfusion injury and cisplatin-induced nephrotoxicity, stress-mediated tiRNAs formation showed in injured renal tissues (42). Notably, ANG overexpression fails to produce tiRNAs under non-stressful conditions, these ANG-overexpressing cells generate significantly more tiRNAs during stress, even mild. This suggests that stress triggers ANG-mediated cleavage of tRNA, which in turn inhibits protein translation (43). Experimental findings demonstrate that tiRNAs mediate stress-related translational suppression by modulating translation initiation factors, including eukaryotic translation initiation factors. This function allows cells to downregulate translation machinery in response to environmental challenges, preserving resources for stress recovery (18). Beyond their role in translational regulation, certain tiRNAs are involved in regulating apoptosis and other key cellular processes. For example, tiRNAs could associate with cytochrome c, thereby inhibiting the apoptotic cascade triggered by the interaction between cytochrome c and APAF-1. ANG mutations reduce tiRNA levels, decreasing their ability to inhibit cytochrome c-mediated apoptosis (44).

### 3.3 RNA-seq and the discovery of tsRNAs

Technological advancements in RNA sequencing enhance detection of functionally significant small non-coding RNAs. Traditional methods construct cDNA libraries by ligating adapters to sncRNA ends followed by reverse transcription. This method is efficient for many sncRNAs with 5'-phosphate and 3'-hydroxyl ends; however, it has inherent limitations (45). For example, some sncRNAs contain specific RNA modifications, including 3'-phosphate or 2',3'-cyclic phosphate groups, which hinder the adapter ligation process. Additionally, RNA methylation modifications, including m1A, m3C, m1G, and m22G, disrupt the process of reverse transcription (46). These modifications significantly impair reverse transcription efficiency and result in incomplete conversion, posing substantial challenges for the detection of both tsRNAs and ribosomal RNA-derived small RNAs. More critically, such technical limitations severely compromise the analytical efficacy for studying cardiovascular diseases associated with specifically modified tsRNAs.

To address these issues, researchers developed PANDORA-seq, which specifically targets modified sncRNAs missed by conventional RNA-seq. This innovative approach employs a dual-enzyme strategy: AlkB family enzymes remove RNA methylation modifications such as m1G, m1A, m3C, and m22G, ensuring efficient reverse transcription, while T4 polynucleotide kinase converts problematic 3'-P or 2',3'-cP to ligation-competent 3'-OH and add a 5'-phosphate end. This method is suitable for sequencing both small and large RNA molecules (8). Traditional single-enzyme treatments are ineffective at capturing modified sncRNAs, while PANDORA-seq optimizes the adapter ligation and reverse transcription process of small RNA fragments (15–50 nt) through sequential enzymatic treatments, significantly improving cDNA library construction efficiency (8).Compared to existing methods, PANDORA-seq demonstrates greater breadth and accuracy in detecting extensively modified sncRNAs within murine and human tissue samples and cellular systems. The advent of this novel high-throughput sequencing technology has overcome the limitations of conventional sequencing approaches, providing unprecedented opportunities for precise profiling of specially modified tsRNAs in cardiovascular diseases.

## 4 Biological function of tsRNA

tsRNAs emerging as versatile regulators of gene expression, far surpassing their original identification as tRNA cleavage byproducts. The varied expression patterns of tRNA genes enable tRNAs to exhibit non-canonical roles beyond their traditional functions (47). Investigations have revealed that tsRNAs are involved in diverse biological pathways, encompassing gene silencing, as well as transcriptional and post-transcriptional modulation, protein translation control, and viral reverse transcription, contributing to diverse physiological and pathological outcomes, including CVDs. The following sections outline the known mechanisms through which tsRNAs exert their regulatory roles.

### 4.1 miRNA-like gene silencing

One of the most well-studied mechanisms of tsRNAs is their capacity to modulate gene expression in a manner analogous to miRNAs, which are small, single-stranded non-coding RNAs, usually ranging from 20 to 25 nt (48). Like miRNAs, tsRNAs engage with Ago proteins to assemble RNA-induced silencing complexes (RISC). These complexes are bound to target mRNAs at the 3' untranslated regions, triggering either inhibition of translation or breakdown of mRNA (49). This miRNA-like behavior is primarily exhibited by tRF-5 and tRF-3 fragments. Research has demonstrated that tRF-3, in combination with Ago3 and Ago4 proteins, forms a silencing complex that can directly bind to the mRNA of targeted genes. This complex enlists mRNA degradation enzymes into specific cytoplasmic processing bodies (P-bodies). Ultimately, this process gives rise to the degradation of the target mRNA, thereby inhibiting the translation of the target gene (4). This miRNA-like behavior is primarily exhibited by tRF-5 and tRF-3 fragments, which can attach to Ago proteins such as Ago1, Ago3, and Ago4, and target mRNAs for silencing (5, 50).

For example, originating from tRNA-Leu and pre-miRNA, tRF/miR-1280 suppresses JAG2, a critical ligand in the Notch pathway, enhancing the progression of colorectal cancer (51). In the uveal melanoma, miRNA and tRF subtype expression levels correlate with diverse molecular phenotypes, metastatic patterns, and survival rates in patients (52). Notably, miR-1247a is mapped to tRNALys3, while miR-1247b aligns with tRNALys5 (53, 54). These two miRNAs exhibit a significant positive correlation with their corresponding tRNAs, indicating a potential correspondence between these miRNAs and tRFs (55, 56). This miRNA-like mechanism provides an avenue for potential therapeutic interventions in diseases where aberrant gene expression plays a critical role, including cardiovascular conditions.

### 4.2 Engagement with RNA-binding proteins (RBP)

tsRNAs modulate gene expressions by engaging with RBPs, key mediators of post-transcriptional regulation by influencing RNA stability, splicing, and translation processes (25, 57). Several tsRNAs, including tRF-Glu, tRF-Asp, and tRF-Gly, bind to YBX1, a typical RBP that modulates mRNA stability and stress responses. These interactions drive cancer progression such as breast cancer by altering transcript stability (26). YBX1 also coordinates stress granule formation and cell survival pathways through associations with 5'tiRNA-Ala and 5'tiRNA-Cys (58).

**Figure 2:**
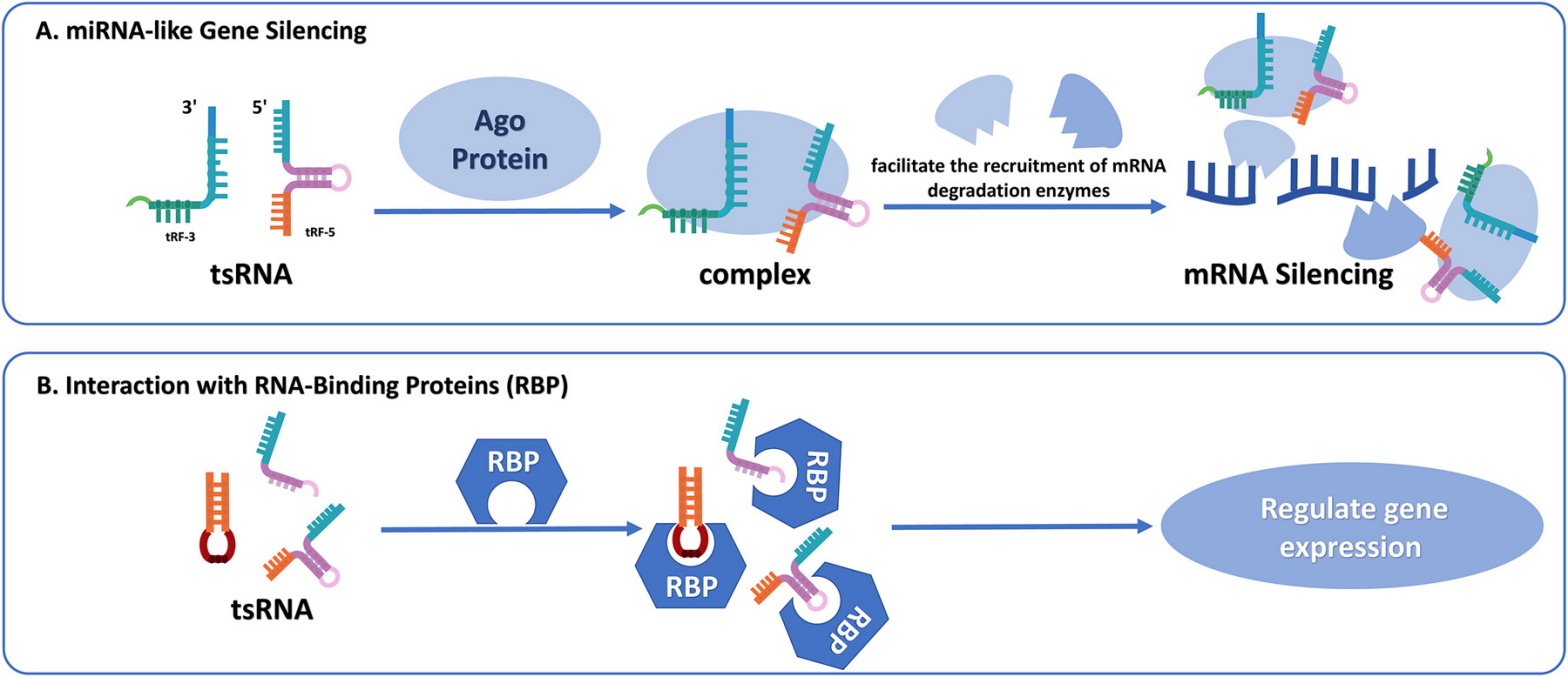
**(A)** tsRNA-mediated miRNA-like gene silencing. Certain tRFs can interact with Ago proteins to assemble RISC. These complexes recognize and bind to complementary target mRNAs, resulting in either inhibition of protein synthesis or transcript destabilization through the recruitment of mRNA degradation enzymes. **(B)** Interaction between tsRNAs and RNA-binding proteins (RBPs). tsRNAs can directly bind to RBPs and influence their stability, localization, or activity. These interactions modulate post-transcriptional gene regulation by affecting mRNA splicing, transport, translation, or decay.

In another example, tRF-2-Ser-TGA binds to the La/SSB (LARP3) protein, stabilizing viral mRNA and potentially contributing to viral replication. This interaction indicates that tsRNAs may be implicated in viral pathogenesis and could be exploited as targets for antiviral therapies (59). These RBP-tsRNA interactions influence diverse cellular processes including cellular growth, adaptation to environmental stressors, and regulation of viral replication. The impact of this RBP-tsRNA combination on CVD-related pathways needs to be further explored (Figure 2).

### 4.3 Regulation of protein translation

**Figure 3:**
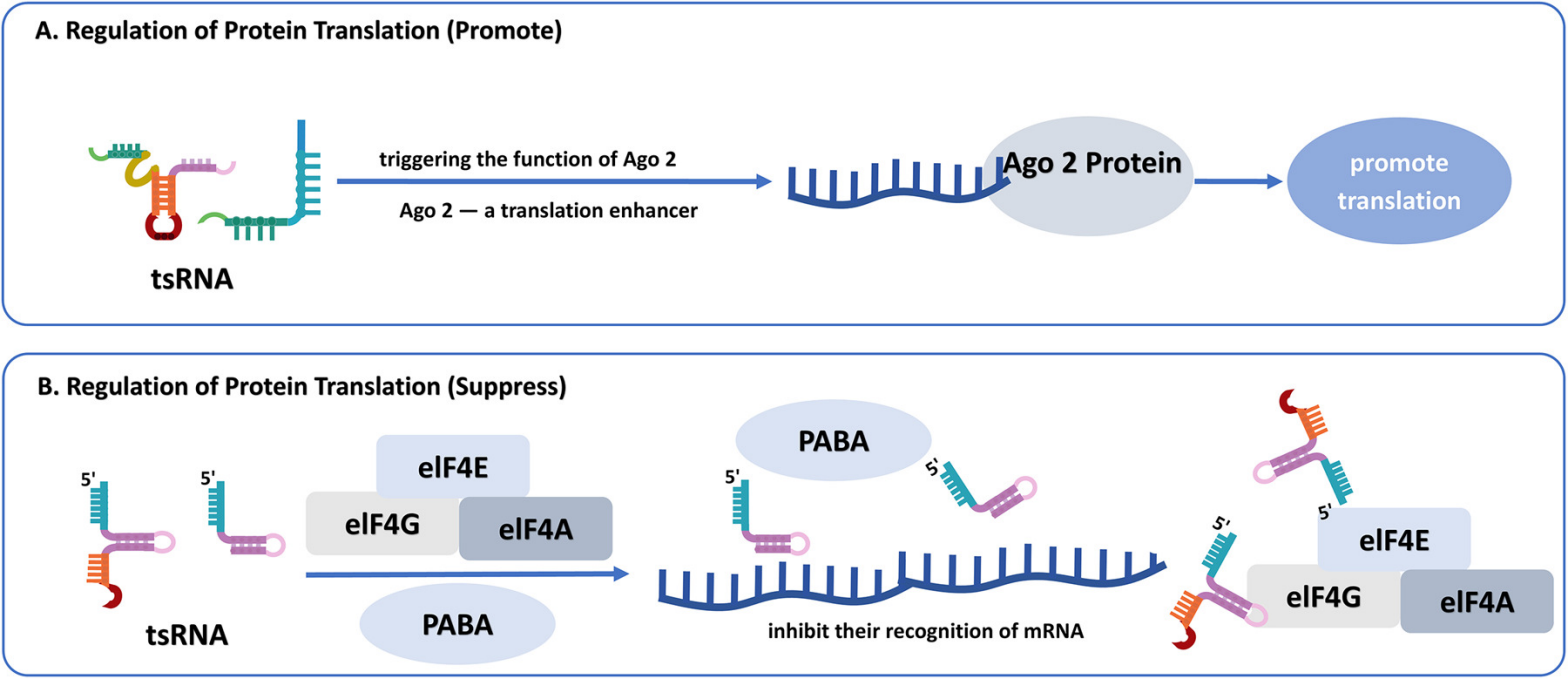
**(A)** The promotive mechanism of tsRNAs on protein translation certain tsRNAs act as molecular switches under specific conditions, triggering argonaute 2 (Ago2), a translation enhancer, thereby facilitating the initiation and enhancing protein synthesis. **(B)** The suppressive mechanism of tsRNAs on protein translation. Certain 5’ tRF fragments inhibit translation initiation by interfering with critical initiation factors, such as eIF4F complex and PABP, thereby reducing mRNA translation efficiency.

tsRNAs bidirectionally regulate protein translation through various mechanisms that correlate to the interaction with AGO. They primarily inhibit translation by interfering with translation initiation. For instance, certain 5' tRFs suppress the initiation of specific mRNAs, including those with a canonical “cap,” a unique “A Cap”, or non-adenylated structures, by interfering with the recognition of mRNA by the eIF4F complex and Poly(A)-binding protein (PABP), critical components of translation initiation (60, 61). Other research illustrates that some tsRNAs with 5'terminal oligoguanine, including 5'tRF-Ala, 5'tRF-Cys, and 5'tRF-Val, can also undergo pseudouridylation modification by PUS7, which enhances their ability to block translation initiation of PABPC1 (62). Furthermore, tRFs and tiRNAs may inhibit protein synthesis by interfering with the assembly of stable, active ribosomes (63). Gebetsberger et al. demonstrated that the halophilic archaeon H. volcanii produces a tRF derived from the 5' fragment of tRNA^Val5^ under certain stress conditions. This tRF binds to the small ribosomal subunit, suppressing peptidyl transferase function and consequently blocking protein synthesis (64). This mechanism highlights the regulatory role of tRFs in modulating protein synthesis under stress conditions. Conversely, some tsRNAs can promote translation. For example, tRF-Leu-CAG enhances the synthesis of RPS28 by modification of mRNA secondary structure, which facilitates access to translation initiation regions (65). Similarly, tRF-Gln19 regulates ribosomal biogenesis by interacting with the multi synthetase complex (MSC), thereby promoting protein synthesis (66). Under stress, tRFs certain tRFs may activate Argonaute 2 (AGO2) to enhance translation (67), suggesting these molecules function as context-dependent regulatory switches (Figure 3).

### 4.4 Regulation of reverse transcription

tsRNAs are critically involved in modulating reverse transcription, particularly during viral replication processes. For instance, in HIV, the tsRNA tRF-3006 derived from tRNA interacts with viral RNA at the primer binding site, acting as a primer for reverse transcription, a critical stage in viral replication (68, 69). Similarly, tRF5-Glu-CTC has been implicated in enhancing the replication of Respiratory Syncytial Virus by binding to the 3'UTR of APOER2, thereby inhibiting its antiviral function and promoting viral replication (69). Also, in HTLV-1, numerous tRFs are expressed, with tRF-3019, originating from the 3' end of tRNA-proline which is the most abundant. While tRNA-Pro typically acts as the reverse transcription primer in HTLV-1, it can be cleaved into tRF-3019, which is shorter but more tightly binding with PBS compared to tRNA-Pro, enhancing the effectiveness and robustness of reverse transcription (70).

**Figure 4:**
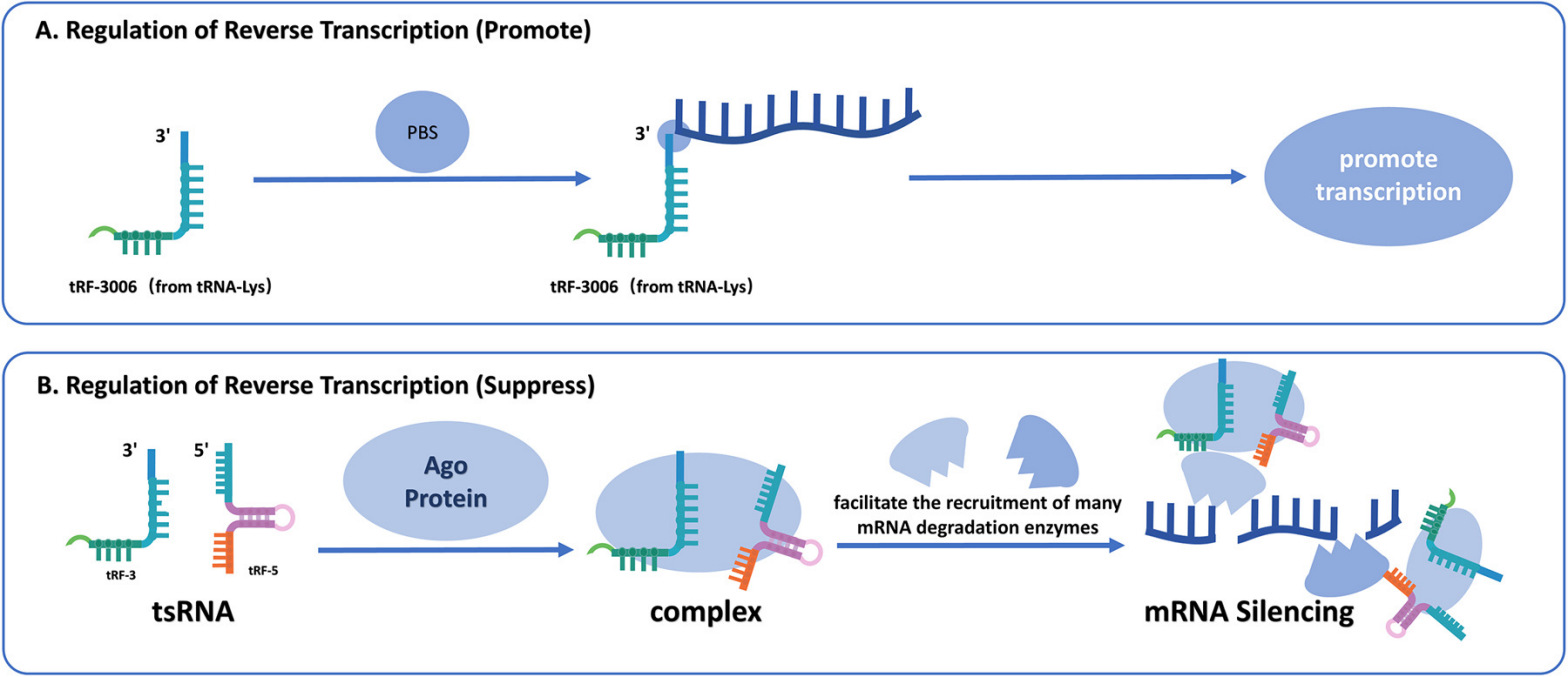
**(A)** The promotive mechanism of tsRNAs on reverse transcription. tRF-3006 derived from tRNA-Lys binds to the PBS on viral RNA, acting as a primer to initiate and enhance the process of reverse transcription. **(B)** The suppressive mechanism of tsRNAs on reverse transcription. tsRNAs (tRF-3 and tRF-5) interact with AGO proteins to form complexes that facilitate recruitment of mRNA-degrading enzymes, ultimately leading to mRNA silencing and inhibition of reverse transcription.

Beyond facilitating reverse transcription, specific tsRNAs can also suppress this process. In the study of the mouse stem cell, abundant tRFs bind to primer binding sites, inhibiting the reverse transcription and mobilization of LTR retrotransposons and endogenous retroviruses, thereby preventing the synthesis of complementary DNA (71). The 22 nt tRF-3b interacts with AGO proteins, directing the RISC complex to target mRNA, resulting in its breakdown or translational inhibition (71). This process underscores the dual functionality of tsRNAs in facilitating and restricting viral replication (Figure 4).

### 4.5 Regulation of rRNA biogenesis

Emerging evidence indicates that tsRNAs may also regulate the biogenesis of rRNA, which is essential for ribosome assembly and protein translation. For instance, tRF-3 has been found to associate with the Twi12-Xrn2 complex (a ribonucleoprotein complex for rRNA processing), an exonuclease responsible for processing precursor rRNA. The tRF-3-Twi12 complex, in coordination with Xrn2 and tRNA-associated protein 1 (Tan1), facilitates the maturation of rRNA, thereby promoting ribosome assembly and ensuring efficient protein synthesis (72–74). Although evidence in this field continues to accumulate, the involvement of tsRNAs in rRNA biogenesis underscores their broader role in regulating cellular homeostasis.

### 4.6 Regulation of cell cycle and apoptosis

**Figure 5:**
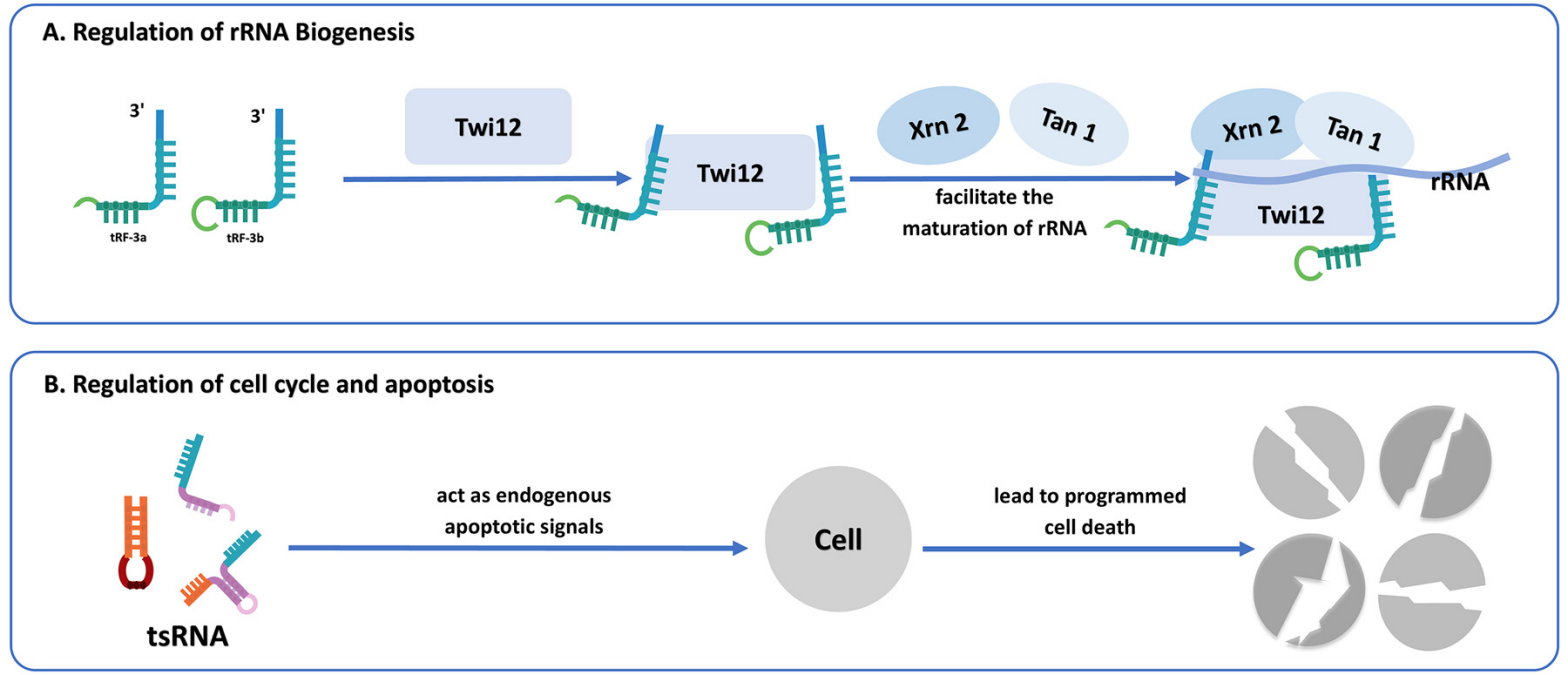
**(A)** The role of tsRNAs in regulating ribosomal RNA biogenesis. tRF-3 interacts with Twi12, Xrn2, and Tan1 proteins, facilitating rRNA maturation and ribosome assembly. **(B)** The regulatory role of tsRNAs in cell cycle and apoptosis. tsRNAs function as endogenous apoptotic signals, leading directly or indirectly to programmed cell death.

Beyond their involvement in RNA silencing and protein synthesis, tRFs play essential roles in regulating programmed cell death. In healthy cells, tRFs function as intrinsic apoptotic signals by suppressing apoptosis-related protein regulators, maintaining homeostasis. However, under stress conditions, tRFs are markedly upregulated, resulting in the evasion of apoptotic regulation and simultaneously inducing the proliferation of malignant cells (66). For instance, depletion of tRF-1001, generated from the 3' end of the Ser-TGA tRNA precursor, results in decreased cell viability, reduced proliferation, and G2 phase arrest (4). Likewise, transfection of NSCLC cells with a tRF-Leu-CAG inhibitor leads to an increase in G0/G1 phase cells and a decline in proliferative capacity (75). Under hyperosmolar stress, tiRNAs can engage with cytochrome c unleashed from the mitochondria *in vivo*. This interaction facilitates the assembly of Cyt c-tiRNA complexes mediated by ANG, which suppress apoptotic body formation under stress, thereby protecting cells from apoptosis (44). Research indicates that the tsRNA-Cyt c complex hinders tsRNA binding to Apaf-1, preventing caspase-9 activation and apoptotic body assembly, thus averting apoptosis (44, 76). Additionally, TRMT10A deficiency, mediated by 5' tRNA^Gln^ fragments (a tRNA methyltransferase), induces pancreatic β-cell death (77) (Figure 5).

### 4.7 New type of epigenetic factors

Epigenetic modifications refer to heritable changes that occur without altering the nucleotide sequence. Emerging evidence positions tsRNAs as potential new epigenetic factors, potentially exerting a substantial influence in the realm of epigenetic mechanisms. Numerous studies have elucidated the functions of tsRNAs in genetics and metabolism, noting that offspring of male mice induced with lipopolysaccharide to develop epididymitis later exhibit metabolic dysfunctions, including impaired glucose tolerance and obesity (78, 79). Further investigation has revealed that sperm cells are rich in tsRNAs, and in the absence of ANG function, the alteration of the sperm tsRNA expression profile triggered by inflammation is effectively prevented, thereby eliminating the metabolic disruptions induced in the offspring by paternal inflammation. Additionally, when 30–40 nt RNA, primarily 5'-tsRNA, extracted from the sperm of male mice with inflammation, is microinjected into normal sperm, it leads to the manifestation of metabolic disorders in the offspring (78, 79).

**Figure 6:**
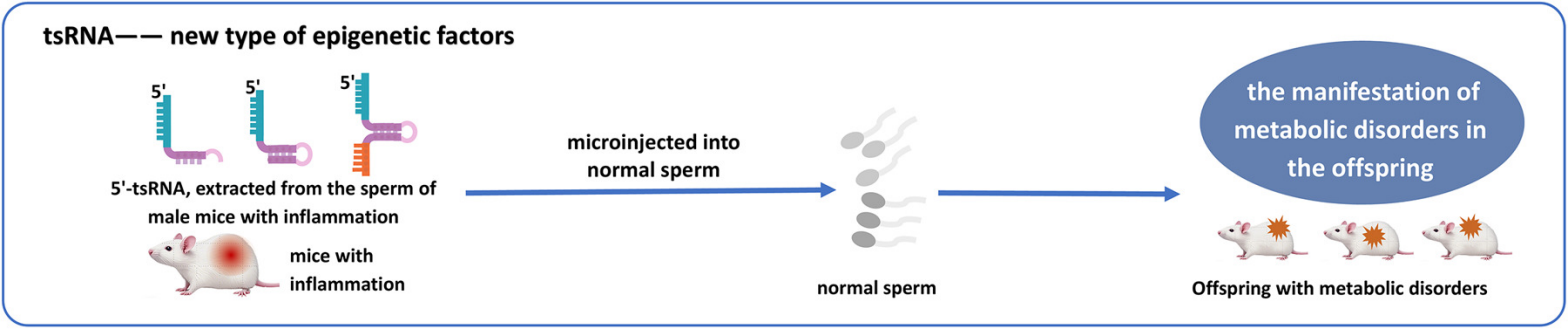
Microinjection of 5'-tsRNAs from inflamed male mice sperm into normal sperm can trigger metabolic dysregulation in offspring, highlighting tsRNAs as epigenetic regulators.

Further mechanistic insights reveal that DNA methyltransferase 2(DNMT2) knockout disrupts sperm tsRNA expression. This aberrant expression is strongly associated with alterations in the mRNA transcriptome of pronuclear embryos derived from wild-type oocytes carrying Kit gene mutations. The knockout of DNMT2 completely blocks the transmission of phenotypic mutations via the oocyte. Further research has demonstrated that both paternal and maternal epigenetic phenotypic inheritance rely on the complete function of DNMT2 in male germ cells (80). This provides a research idea for further study of the potential of tsRNA as a novel epigenetic factor (Figure 6).

## 5 Potential effects of tsRNAs in CVDs

CVDs comprise a spectrum of pathological conditions impacting cardiac and vascular system, representing a worldwide public health challenge (81). In 2022, CVDs accounted for approximately 1.9 million deaths worldwide (81), with pathologies including myocardial ischemia, cardiac hypertrophy, atherosclerosis, varicose veins, and pulmonary arterial hypertension, among other clinical manifestations. Despite notable advancements in diagnostic and therapeutic technologies, the molecular mechanisms underlying the homeostasis of cardiomyocytes in the development and progression of CVDs remain incompletely elucidated (82), a gap in knowledge that limits the development of more effective treatment strategies. Recent research has demonstrated the involvement of tsRNAs in the pathophysiological processes of CVDs through gene expression modulation (83). Owing to their exceptional detection accuracy and molecular stability in biofluids including plasma and exosomes, these make them not only serve as optimal noninvasive indicators for disease detection and outcome prediction but also provide new research directions for the development of precision medicine (84) (Table 1).

**Table 1:** Functional roles and mechanisms of tsRNAs in CVDs.

Cardiovascular disease type	tsRNA [example]	Key function	Mechanism	Disease model	Reference
Atherosclerosis	tsRNA-21	Early biomarker for coronary events	Early diagnostic biomarker	Plasma from coronary artery disease patients	(110)
Heart failure	tRF-60:76-Val-AAC-1-M5	Prediction of drug response	Sacubitril/valsartan resistance regulation	Peripheral blood (HF patients on sacubitril/valsartan)	(95)
Heart failure	tRF-Tyr-GTA-010/tRF-Tyr-GTA-011	Regulation of calcium signaling for cardiac protection	Sphingolipid, adrenergic, and calcium signaling	Mouse heart tissue (HF model)	(85)
Cardiac hypertrophy	tRF-16-R29P4PE	Regulation of cardiac metabolism and hypertrophy	PACE4 and metabolic signaling	Human plasma; H9c2 cells; Sprague-Dawley rats	(122)
Myocardial infarction	tsRNA-0406	Prevention of excessive scar formation.	ceRNA-mediated extracellular matrix regulation（ECM）	MI mouse model; neonatal mouse cardiomyocytes	(85)
Myocardial infarction	tsr007330	Regulation of myocardial fibrosis	Inhibition of NAT10/EGR3-mediated fibrosis	MI rat model	(94)
Myocardial infarction	5'tRF-LysCTT	Regulation of ferroptosis	Promotes ferroptosis	MI mouse model, H9c2 cardiomyocytes	(93)
Myocardial infarction	HC83	Protection of cardiomyocytes	Regulation of MIAT/VEGFA and mitochondrial integrity	MI mouse model; neonatal mouse cardiomyocytes	(95)
Myocardial infarction	tiRNA-Val-AAC-2-32/tiRNA-Lys-TTT-1-34	Early diagnosis of myocardial infarction	Early plasma biomarker	Human plasma samples	(92)
Myocardial infarction	tRF-50304	Promotion of cardiomyocyte apoptosis	Pro-apoptotic regulation under hypoxia	AC16 cardiomyocytes, H9c2 cells	(90)
Myocardial infarction	tRF5-22-SerGCT-1	Protection of cardiomyocytes	Inhibition of MSK1; modulation of MAPK and autophagy pathways	MI mouse model; OGD-treated cardiomyocytes	(97)
Fulminant myocarditis	tiRNA-Gln-TTG-001	Early diagnosis of acute inflammatory cardiomyopathy	Acute-phase biomarker for FM	Coxsackievirus B3-induced FM mouse model; serum from FM patients	(113)
Atherosclerosis	tsRNA-5001a	Enhancement of plaque stability	m6A-mediated BCL2 regulation for plaque stability	ApoE knockout mice; vascular smooth muscle cells	(114)
Atherosclerosis	tsRNA-0420	Contribution to plaque instability	Activation of NLRP3 inflammasome	ApoE knockout mice; RAW264.7 macrophages	(86)
Atherosclerosis	tRF-2	Loss of protective expression in normal vessels	Marker of vascular homeostasis	ApoE knockout mice; human vascular tissue samples	(108)
Atherosclerosis	tRF-3b	Downregulation in disease progression	Loss of vascular protective function	ApoE knockout mice; human vascular tissue samples	(108)
Atherosclerosis	tRF-5a	Upregulation in disease progression	Promotion of vascular dysfunction	ApoE knockout mice; human vascular tissue samples	(108)
Atherosclerosis	tiRNA-Glu-CTC	Induction of VSMC phenotypic switching and vascular injury	Cacna1f downregulation and mitochondrial damage	Vascular smooth muscle cells (*in vitro*)	(115)

### 5.1 tsRNA and MI

MI, clinically termed as heart attack, arises from the blockage of a coronary artery, resulting in diminished blood flow and oxygen delivery to the cardiac muscle. This ischemic event results in cardiomyocyte death and tissue necrosis, contributing to impaired heart function. The pathogenesis of MI is often described in three distinct stages: (1) Atherosclerotic plaque rupture—A plaque in the coronary artery, composed of lipids and fibrous tissue, ruptures, triggering thrombus formation and occlusion of the vessel; (2) Ischemia-reperfusion injury—Reperfusion induces oxidative stress and mitochondrial damage, which exacerbate cellular injury damage via reactive oxygen species(ROS) generation, necessitating cardiomyocyte necrosis and programmed cell death; (3) Maladaptive cardiac remodeling—Post-infarction, the heart undergoes structural changes including fibrosis, which reduces contractile function and increases the risk of heart failure (65). Although clinical markers such as cardiac troponins are utilized for MI diagnosis, they exhibit delayed elevation post-infarction, limiting early detection (85). Further understanding of the molecular mechanisms of MI opens the door to studying diagnostic and therapeutic strategies for non-coding RNAs.

Evidence indicates the decisive role of tsRNAs in the pathophysiology of MI. Under pathological stimuli including ischemia and oxidative stress, tRNA undergoes cleavage, generating tRF and tiRNA fragments. These RNA fragments modulate multiple signaling cascades related to cell survival, programmed cell death, and inflammatory processes (86). Clinically, tsRNAs present in cellular and plasma samples as distinctive biomarkers of cellular stress status. These small RNAs exhibit dynamic changes after cardiopulmonary bypass (CPB) cardiac surgery. Experimental evidence reveals that cardiac fibroblasts and cardiomyocytes secrete 4,025 and 3,500 extracellular tsRNAs respectively during nutrient deprivation, while releasing 3,931 and 5,259 distinct tsRNA species under oxygen-deficient conditions. Importantly, 122 tsRNAs show significant changes in expression after CPB surgery, with 41 upregulated in-patient plasma (87). In ischemic cardiomyocytes, the expression pattern of tsRNAs undergoes significant changes. Dhahbi et al. discovered age-dependent fluctuations in 5' tiRNAs in mouse serum, which were regulated by caloric restriction (CR) (88). Sequencing analysis revealed that during myocardial ischemia, 166 tsRNAs were upregulated, while 136 tsRNAs were downregulated, suggesting that these molecules may participate in the cardioprotective mechanisms of CR and mediate ischemic stress adaptation and myocardial tissue remodeling. Specifically, in CR-treated mice, cardiac tissue exhibited upregulation of tRNA-His-GTG-004 and downregulation of tRF-Cys-GCA-022, tRF-Lys-CTT-026, and tRF-Met-CAT-008. These dysregulated tsRNAs potentially mediate therapeutic outcomes through modulation of specific genetic targets including Med13l, Sucla2, and Wls (89). Temporal regulation is evident as 5' tRNA-Val-ACC peaks 3 h post-ischemia (7).

Specifically, tsRNAs regulate cardiomyocyte death by regulating pathways such as apoptosis, necroptosis, and autophagy, which as crucial mechanisms in MI pathogenesis. For example, tsRNAs (such as tRF-50304) have been identified to bind to specific mRNA targets, promoting the stability of pro-apoptotic genes and exacerbating cardiomyocyte death under hypoxic conditions (90). Additionally, certain tiRNAs, such as 5'-tiRNA-Gly-CCC, are associated with platelet activation and thrombus formation, two key events that exacerbate ischemic injury (91). In the process of fibrosis and remodeling after tsRNAs regulate MI, with fragments such as tsRNA-0406 acting as competitive endogenous RNAs to regulate extracellular matrix proteins and prevent excessive scarring (85). In the mouse model of AMI, tiRNA-Val-AAC-2-32 and tiRNA-Lys-TTT-1-34 are specifically elevated in plasma early after infarction, with tiRNA-Lys-TTT-1-34 increasing as early as 6 h after injury, indicating their potential diagnostic relevance (92).

tsRNAs not only have potential diagnostic value but also demonstrate novel therapeutic strategies in MI or myocardial injury. For instance, 5'tRF-LysCTT is associated with ischemia-reperfusion injury, and its knockdown can alleviate H/R-induced cardiomyocyte ferroptosis, while its overexpression exacerbates cell death. This suggests that 5'tRF-LysCTT represents a promising druggable target for managing myocardial ischemia-reperfusion damage (93). The tsRNA tsr007330 is significantly downregulated after MI, and by antagonizing N-acetyltransferase NAT10, it inhibits the ac4C modification of early growth response protein 3 (EGR3) mRNA mediated by NAT10, thereby reducing myocardial fibrosis and improving cardiac function. This regulator*y* axis (tsr007330/NAT10/EGR3) reveals a new mechanism for tsRNA regulation in myocardial fibrosis after MI, providing a new direction for targeted intervention (94). Comprehensive profiling of epicardial adipose tissue from heart failure cases identified 343 distinct tsRNA species, among which 24 exhibited marked differential expression patterns. Distinct fragments including tRF-Tyr-GTA-010 and tRF-Tyr-GTA-011 could potentially preserve cardioprotective function by modulating calcium homeostasis through sphingolipid metabolic pathways and adrenergic receptor signaling. Computational biology analyses indicate these tsRNAs could participate in molecular pathways associated with heart failure development (85). Notably, tsRNAs can be used to predict drug treatment responses, particularly in post-MI heart failure cases. Elevated tRF-60:76-Val-AAC-1-M5 levels correlate significantly with treatment responsiveness, with ROC curve validation confirming its robust predictive capacity. This tsRNA may affect treatment response by regulating lipid metabolism and apoptosis-related genes, such as Tnfrsf10b and Bcl2l1, suggesting its potential as a marker for therapeutic efficacy (95). Additionally, the tRNA-derived fragment HC83 from the traditional Chinese medicine ginseng shows significant cardioprotective effects in ischemia/reperfusion injury models. By targeting lncRNA MIAT, it upregulates VEGFA expression, improves mitochondrial function and cytoskeletal stability, and significantly promotes cardiomyocyte survival. Animal experiments show that the protective effect of HC83-mimic is far stronger than metoprolol, suggesting that plant-derived tsRNAs may become a new direction for RNA therapy (95). tsRNAs may contribute to therapeutic variability in sacubitril/valsartan treatments among post-MI heart failure patients (96). The tsRNA sRNA-04002 regulates endothelial-mesenchymal transition to prevent excessive fibrosis and vascular remodeling after cardiac injury (86). In addition, a recent study identified tRF5–22-SerGCT-1 as a cardioprotective tsRNA that targets MSK1, thereby modulating MAPK and autophagy pathways to mitigate myocardial injury in MI mouse models and OGD-treated cardiomyocytes (97). These discoveries demonstrate the dual utility of tsRNAs as both diagnostic indicators and therapeutic candidates for MI, providing new approaches for interventions to improve myocardial recovery after MI and prevent further damage.

### 5.2 tsRNA and atherosclerosis

Atherosclerosis represents a chronic inflammatory condition affecting the arterial walls, marked by lipids deposition, immune cell infiltration, fibrous tissue expansion and atherosclerotic plaques formation (98). This process begins with endothelial dysfunction triggered by various risk factors, including elevated blood pressure, hyperlipidemia, and smoking. These factors cause endothelial cells to become more permeable, allowing LDL particles to infiltrate the intima. LDL retention and oxidation exacerbate further inflammation via endothelial activation and monocyte recruitment, with subsequent differentiation into macrophages. These phagocytes internalize oxidized LDL, transforming into lipid-accumulating foam cells that constitute the lipid-laden core of plaques (99–102). During plaque development, smooth muscle cells undergo trans-migration into the intimal layer, where cellular proliferation and extracellular matrix deposition contribute to fibrous cap formation (103). However, plaque rupture typically occurs when the fibrous cap weakens, a process closely linked to matrix metalloproteinase (MMP) activity. MMP-1, MMP-8, and MMP-13 contribute to collagen degradation within the fibrous cap, thinning its structure and increasing the risk of rupture. Additionally, MMP-2 and MMP-9 promote intraplaque neovascularization, further destabilizing the plaque. Once a rupture occurs, exposure of prothrombotic factors triggers thrombosis, which may lead to MI or stroke (104). NF-*κ*B and MAPK signaling pathways critically regulate this oxidative stress and inflammatory cascade (105).

tsRNAs (tRFs and tiRNAs) have emerged as significant modulators of gene expression in cardiovascular pathologies, such as atherosclerosis. Produced from mature or precursor tRNAs during cellular stress or cellular injury, these sncRNAs are instrumental in modulating key cellular processes, including inflammation, lipid metabolism, and cellular proliferation (18, 106). Recent studies demonstrated differences in tsRNA expression between atherosclerotic vascular tissues and normal vessels. Specifically, 315 tsRNAs exhibited altered expression in atherosclerotic vessels, with 131 upregulated and 184 downregulated (107). Among them, tRF-2 was absent in atherosclerotic tissues but present in healthy tissue, while tRF-3b expression was markedly reduced, and tRF-5a was notably upregulated (108). Notably, recent research has demonstrated that overexpression of tRF-5cs, tRFGly-GCC-009, and tRF-Gly-GCC-008, as well as down-regulation of tRF-Pro-AGG-006 and tRF-Pro-AGG-005, were detected in tissue samples of AS patients relative to healthy controls, implying their potential role in AS-associated pathological signaling pathways (109). Experimental analyses revealed that the upregulation of tRF-Gly-GCC-009 may induce abnormal cellular adhesion dysfunction and contribute to the development of AS. Furthermore, this class of tsRNAs demonstrates significant associations with the Apelin pathway, calcium signaling, and Notch signaling pathways. Nevertheless, the exact biological mechanisms governing these regulatory effects remain to be fully elucidated (109). *in vitro* experiments have substantiated that elevated expression of tRF-Gly-GCC proliferation and migration of VSMCs and boost monocyte adhesion to endothelial cells, potentially facilitating atherogenesis (108). Increased concentrations of tiRNA-Gly-GCC have been identified in both vascular tissues and plasma of atherosclerosis patients, suggesting its prospective as an innovative biomarker and therapeutic target for atherosclerosis (110). Moreover, tsRNA-21 has been demonstrated to have a strong correlation with early coronary artery calcification, showing 92% sensitivity and 88% specificity in plasma samples (110). A novel high-throughput sequencing method, PANDORA-Seq, was employed to analyze the intimal tissue of LDLR^−^^/^^−^ mouse atherosclerosis models, identifying 195 differentially expressed tsRNAs. Among these, tsRNA-Arg-CCG was identified as a potential regulator of pro-atherosclerotic gene expression, possibly by acting on vascular endothelial cells to promote lesion formation (111). Notably, HDL-associated m^1^A-tDR-ArgACG-1 activates macrophage adhesion and pro-inflammatory responses through the SR-BI-mediated signaling pathway, independently of cholesterol efflux, suggesting its potential involvement in atherogenesis (112). Comparative analysis reveals differential expression profiles of tRF-Gly-GCC and tRF-Pro-AGG in atherosclerotic plaques vs. normal carotid tissues, implicating their contribution to disease pathogenesis (113). For instance, tsRNAs such as tsRNA-0420, which is derived from platelet-derived RNA, have been implicated in the induction of the NLRP3 inflammasome in macrophages, amplifying the inflammatory response and enhancing the secretion of IL-1β, a cytokine that contributes to plaque instability (86). tsRNAs can modulate the phenotypic transition of VSMCs, a critical mechanism influencing plaque stability. For instance, tsRNA-5001a, enriched in VSMCs, has been found to inhibit apoptosis and promote the formation of a stable fibrous cap by modulating BCL2 expression through m6A modification, thus contributing to plaque stability (114). Emerging evidence indicates that nanoplastics exposure triggers the overexpression of tiRNA-Glu-CTC, which facilitates the transition of VSMC from contractile to synthetic phenotype. This transformation is accompanied by mitochondrial dysfunction, ROS accumulation and dysregulation of calcium signaling, thereby exacerbating vascular injury. This tiRNA exerts its effects by regulating the expression of Cacna1f, indicating that it may function as a novel intervention target for environmentally induced CVDs (115). These findings collectively establish tsRNAs as both diagnostic markers and therapeutic targets for atherosclerosis management.

### 5.3 tsRNA and cardiac hypertrophy

Myocardial hypertrophy represents an adaptive alteration in response to physiological or pathological stimuli, increasing cardiomyocyte size rather than number. Pathological myocardial hypertrophy can lead to heart failure through myocardial remodeling, serving as a primary determinant of elevated disease burden and mortality in aging population (116). Both nuclear-encoded and mitochondria-derived tsRNAs exhibit altered expression during this pathological process, suggesting a close relationship between tsRNAs and myocardial hypertrophy.

Nuclear-encoded tsRNAs participate in the modulation of cardiac hypertrophy, particularly in response to hypertrophic stimuli and oxidative stress (86). In isoproterenol-induced hypertrophic myocardium, tRF molecules like tRF-1 tRNA-Gly-CCC exhibit significantly elevated expression levels (117). Mechanistically, these tRF molecules can mimic miRNA functions by specifically targeting the 3' UTR of the Timp3 gene to regulate myocardial hypertrophy progression. Experimental evidence confirms that overexpression of tRFS1 and tRFS2 enhances the expression of cardiac hypertrophy markers, including ANF, BNP, and β-MHC (118). Oxidative stress-related tsRNAs, such as Val-and Gly-5'-tiRNA, are enriched in hypertrophic cardiomyocytes, indicating their role in stress adaptation (88). Previous research has also shown that tRFs in paternal germ cells can affect cardiovascular development in offspring through epigenetic modifications, contributing to heritable cardiac hypertrophy (119). In mouse fibroblasts, upregulated tsRNAs competitively interact with cytochrome c, disrupting its association with apoptotic protease-activating factor-1. This process inhibits apoptosome assembly and programmed cell death via activation of caspase-9 and caspase-3 (120). Compared to hyperosmotic conditions, oxidative stress activates more tiRNAs, with enhanced binding to cytochrome c resulting in decreased availability of apoptosis-inducing free cytochrome c (37).

Beyond nuclear tsRNAs, mt-tRFs have been linked to genetically inherited forms of cardiac hypertrophy. The m.3243A > G mutation in mitochondrial tRNA-Leu-UUR, clinically linked to mitochondrial encephalomyopathy, lactic acidosis, and stroke-like episodes, has been shown to alter mt-tRF expression, potentially affecting mitochondrial function in hypertrophic cardiomyopathy(HCM) (88). ELAC2 mutations have been found to disrupt mt-tRF biogenesis, contributing to infantile HCM (121). Furthermore, tRF-16-R29P4PE is significantly downregulated in patients with pathological cardiac hypertrophy, demonstrating its potential as a biomarker. Research indicates that this tsRNA regulates cardiomyocyte metabolism and mitochondrial function by modulating the PACE4 and HIF-1α/PPARα signaling pathways, thereby attenuating the hypertrophic phenotype. Targeting tRF-16-R29P4PE or its downstream signaling pathways may offer novel intervention strategies for treating cardiac hypertrophy and associated metabolic disorders. These findings position tRF-16-R29P4PE as a potential therapeutic candidate for attenuating pathological cardiac hypertrophy progression and associated metabolic dysregulation (122).

### 5.4 tsRNA and aortic dissection

Aortic dissection (AD) stands as a cardiovascular catastrophe exhibiting low prevalence yet exceptionally high mortality. Its pathogenesis involves the intricate three-layer structure of the aortic wall—intima, media, and adventitia. AD is characterized by intimal tearing, allowing blood to flow into the media, forming true and false lumens, typically accompanied by severe tearing pain at onset (123). The underlying pathological mechanisms encompass complex, multifactorial processes involving dynamic interplay between diverse cellular components and the extracellular matrix elements (124).The development of AD is closely linked to the activity of inflammatory cells, including lymphocytes and macrophages. Throughout this process, there is an upregulation of protease and calmodulin expression, accompanied by the release of ROS. These factors contribute to the apoptosis of VSMCs, which in turn lead to the rupture of AD (123, 125). Immune cell infiltration into the aortic wall constitutes a defining pathological feature. Macrophages orchestrate inflammatory responses by recruiting various immune cell populations, mediating essential regulatory functions in disease progression (124).

In the study of AD, Zong et al. observed a significant reduction in 5'tiRNA-Cys-GCA levels in a mouse AD model. This molecule is regarded as a potential modulator of phenotypic transition, as its overexpression inhibits VSMCs proliferation and migration while elevating α-smooth muscle actin expression (126). Investigations further reveal that oxidized LDL stimulation upregulates STAT4, a crucial transcription factor directly modulated by 5'tiRNA-Cys-GCA, thereby enhancing cellular proliferation, motility and phenotypic switching. However, this process can be reversed by the overexpression of 5'tiRNA-Cys-GCA. Treatment with 5'tiRNA-Cys-GCA reduces the incidence of AD in mice induced by angiotensin II and β-aminopropionitrile, as well as prevents its malignant progression (126).

Fu et al. conducted research shown that comparison of aortic tissues from AD patients with healthy controls, revealing significant dysregulation of tsRNAs in AD samples, with tRF-1:30-chrM.Met-CAT showing marked upregulation (127). *in vitro* studies confirmed this tsRNA enhanced VSMC proliferation, migration, and phenotypic transition. Investigators compared tsRNA expression patterns between quiescent and proliferating human aortic smooth muscle cells (HASMCs), finding that two tsRNAs promoted aortic smooth muscle proliferation through different mechanisms: AStDR-000067 promotes HASMC proliferation by inhibiting p53 transcription through binding to the promoter region, while AS-tDR-000076 accelerates HASMC proliferation by targeting the 3'-UTR to reduce MFN2 levels (128). Li et al. have developed an innovative gene therapy approach for aortic dissection/aneurysm (AAD) by leveraging the role of tsRNAs to modulate VSMC functionality and inflammatory cell regulation. They employed neutrophil membrane-mimetic nanovesicles encapsulating therapeutically active tRF-Gly-CCC as a delivery platform. The results indicate that novel microvesicles display superior stability in the circulatory system, achieving precise localization to aortic lesion and significantly decreasing mortality in acute aortic dissection cases. This approach illuminates the therapeutic potential of tsRNAs for AAD, offering a promising avenue for early intervention and improved clinical outcomes (129). In conclusion, a variety of tsRNAs participate in VSMC proliferation and motility through different mechanisms, and have significant contributions to the occurrence and progression of AD.

### 5.5 tsRNA and pulmonary arterial hypertension

Idiopathic pulmonary hypertension (PAH) represents a lethal pulmonary vascular disorder causing progressive vascular remodeling, pulmonary artery occlusion, and elevated pulmonary vascular resistance (130). Pathophysiologically, idiopathic PAH demonstrates cancer-like properties, featuring dysregulated cellular metabolism, aberrant proliferation, and apoptotic resistance (131). Better treatments are urgently needed despite recent advances. tsRNAs appear to influence PAH through multiple mechanisms. Researchers identified tsRNA expression patterns in murine circulatory, right ventricular tissue, and lung samples by establishing a monocrotaline-induced pulmonary hypertension rodent model, while comparing plasma samples from PAH patients with healthy individuals. Analytical results included identification of 2,716 unique tsRNA species in human plasma vs. 4,733 in rodent tissues, demonstrating a 7.84% concordance rate. Also, 204 tsRNAs showed high conservation across all sample types, indicating their potential critical regulatory functions in PAH pathogenesis (132).

A separate small RNA microarray study comparing idiopathic PAH patients with healthy controls revealed 816 differentially expressed tsRNAs, with 243 upregulated and 573 downregulated. Real-time qPCR confirmed the differential expression of eight tsRNAs, including four upregulated (such as tRF3a-AspGTC-9, 5'tiRNA-31-GluCTC-16) and four downregulated (such as 5'tiRNA-33-LysTTT-4, i-tRF-8:32-Val-AAC-2). Bioinformatics analysis suggested these tsRNAs potentially contribute to PAH development through modulation of critical genes including BMPR2 and AQP1. Notably, i-tRF-31:54-Val-CAC-1 may promote the progression of PAH by targeting BMPR2 (133).

Studies demonstrated that angiopoietin, a newly discovered mediator of pulmonary hypertension, dose-dependently upregulated tRFs, including 5-tRF-Gly-GCC and tRF-Glu-CTC and accelerated cell death (134). In a PAH rat model, ANG modulates endothelial apoptosis via the ANG-tsRNAs-caspase-3 axis with a sponge effect (44). Mitochondrial tsRNAs (such as mt-i-tRF-Glu-UUC) exhibited significant expression abnormalities in PAH patients, further supporting the association between tsRNAs and PAH pathogenesis (135). These insights reveal promising therapeutic targets for PAH management.

### 5.6 tsRNA and other CVDs

Fulminant myocarditis (FM) is an uncommon yet rapidly progressing inflammatory cardiac condition, characterized by fast progression and high mortality rate (136). While endomyocardial biopsy remains the diagnostic criterion standard, its invasive nature renders the procedure intolerable for many patients (137). There is currently a lack of sensitive biomarkers for early assessment of disease severity and prognosis. Through small RNA sequencing plasma samples from pediatric FM cases during acute/recovery phases and healthy controls revealed markedly elevated tiRNA-Gln-TTG-001 levels in acute FM. *in vitro* experiments showed a significant increase in its generation and extracellular release, suggesting diagnostic utility (113).

In calcific aortic valve disease (CAVD), a condition associated with metabolic disorders, abnormal lipid metabolism and Ca^2^⁺ deposition initiate the disease, which often coexists with atherosclerosis and aortic stenosis (138). Research has identified tsRNA-5006c (5'tRF-Lys-CTT) as possessing distinctive biological attributes. This tsRNA is detectable in extracellular vesicles from M1-polarized macrophages and can be transported to aortic valve interstitial cells (AVICs), where it acts as a “messenger” to transmit signals. This signaling promotes mitotic activity and enhances osteogenic differentiation in AVICs during CAVD pathogenesis (139). Future research may explore the modulation of tsRNA-5006c expression or activity blocking it signaling in AVICs, thereby intervening in the abnormal mitotic and osteogenic differentiation processes and achieving therapeutic goals for CAVD (139). However, a comprehensive understanding of this pathogenic mechanism is still required before specific interventions can be effectively implemented.

Investigations in rheumatic heart disease populations demonstrated marked dysregulation of tsRNA expression profiles in AF patients compared to sinus rhythm counterparts, irrespective of AF comorbidity status. Using high-throughput sequencing, 219 accurately matched tsRNAs were determined in three pairs of cardiac papillary muscles, with 77 tsRNAs showing marked differential expression between the two groups (140). Further analysis indicated that AS-TDR-001269, AS-TDR-001363, and AS-TDR-006049 were the most prominently differentially expressed tsRNAs. Bioinformatic analysis indicated that these differentially expressed genes participate in essential biological pathways including transcriptional modulation, DNA binding, and intracellular transcriptional regulation. Gene enrichment analysis predicted that most of these genes are closely related to interactions with cytokine receptors, especially the targeting relationship between chemokine ligand 5 (CCL5) and AS-tDR-001363. In patients with RHD and AF, CCL5 expression was significantly reduced. Further experiments showed that CCL5 expression levels were reduced in AC16 cells transfected with AS-tDR-001363 (140). Significant abnormalities in tsRNA expression have been observed in age-related atrial fibrillation (AF). Research indicates that curcumin attenuates oxidative stress and inflammation levels while ameliorating atrial fibrosis, ultimately decreases atrial fibrillation susceptibility in aged murine through downregulation of the specific mature mt-tRNA-Val-TAC-CCA termini in cardiac atria. This finding suggests the potential for specific tsRNAs to be targeted as an intervention strategy in age-related AF. The modulation of these tsRNAs could offer a novel approach to mitigating the progression of AF in older populations (141). These results elucidate the molecular pathogenesis of AF secondary to RHD and identify targets for its management and therapeutic intervention.

## 6 Conclusions

As an emerging class of non-coding RNAs, tsRNAs can be categorized into tRFs and tiRNAs, distinguished by their distinct biogenesis pathways. These molecules are produced through endonucleolytic processing of either mature tRNAs or precursor tRNA transcripts by specialized ribonucleases. These molecules participate extensively in fundamental cellular activities, including gene transcription regulation, translational repression, stress response, and epigenetic modification, and have demonstrated complex and diverse regulatory functions, especially in CVD. This review synthesizes recent advances in understanding tsRNAs within CVD, elucidating their molecular functions and regulatory mechanisms. In CVD pathogenesis, specific tsRNA subtypes exhibit bidirectional roles in various cardiac pathologies, including MI, atherosclerosis, and cardiac hypertrophy by regulating VSMC proliferation, inflammatory responses, apoptosis, and fibrosis through mechanisms that may either exacerbate injury or promote repair, with specific effects depending on subtype, target, and microenvironmental conditions. Although much attention has been paid to the diagnostic potential (e.g., as early biomarkers) and therapeutic value (e.g., targeted intervention in inflammation and fibrosis) of tsRNAs in CVD, there are still many blind spots in their mechanism of action. For example, the interaction network between tsRNAs and specific proteins, the specific pathways of epigenetic regulation, and the spatial and temporal expression characteristics of different isoforms in the disease have not been fully elucidated. In addition, its clinical application still faces technical challenges, including standardization of detection methods, efficient capture of modified RNA, and optimization of delivery systems. In the future, researchers should combine multi-omics techniques, animal models and clinical cohorts to systematically analyze the dynamic regulatory network of tsRNAs and explore their application in precision medicine, to open new avenues for the prevention and treatment of CVDs.
